# Remounts, U. S. Army

**Published:** 1890-02

**Authors:** Wm. Baird

**Affiliations:** 6th Cavalry, U. S. A.


### REMOUNTS, U. S. ARMY.

Abstract from Article "How to Get There."

BY FIRST-LIEUTENANT WM. BAIRD.

6th Cavalry, U. S. A.

[From the *Journal of the U. S. Cavalry Association*, December, 1889.]

WHILE there is little or no dissent from the idea that our present way of getting horses for the service can be improved, the methods proposed differ widely. The importance of the subject and the desirability of making even the smallest steps in advance, should be a sufficient appeal for us to lay aside elaborate plans that seem unlikley to be carried out at present, and unite in working for what can probably be obtained to start with. For if the results are but slight at first, evidence of their being in the right direction will ensure our obtaining further and further concessions, and in the end full realization.

Under a mode recently followed for a few years, a contract is made with the lowest bidder at some particular place at Louisville, St. Louis, etc., to furnish a certain number. Horses are presented to be inspected by a *civilian* hired for the purpose by the Quartermaster's Department, and if "passed" the money is paid by an officer of the Quartermaster's Department.

Some of the defects inherent in this method are :—

*First.* The successful bidder fills his contract by sub-letting, or else reaches the distant portions of territory from which he draws, by sub-agents, middlemen or minor dealers. Take out their various profits, also the expense of bringing the stock in to the point of delivery, it is easily seen that the figure offered the breeder must be so low as to purchase only a class of horses inferior to what is attainable at the contract price.

It may be replied, that horses delivered or offered for sale at a central point like St. Louis, must be a little higher than if sought out individually on the farm ; but this is one of the unnecessary expenses of the system, the remedy for which will be spoken of later on.

*Second. There is almost nothing to be done when new horses with disqualifying defects are finally received by the troop commander.* They are often purchased just before or just after the new fiscal year begins, then banged around in cars and stockyards for days and nights of mid-Summer heat and thirst ; and possibly then herded and driven over miles of dry desert or alkaline plains. Every batch of new, green horses must have on arrival some which are out of condition, sick with distempers and other disorders, or injured in sight, head or limb. An examining board at this stage is hardly ever able to accomplish much. The present system does not seem to contemplate any inspection ; but should a board be convened to act on certain horses, any defect will be alleged to have been entirely produced *en route*, or, anyhow, increased to a disqualifying degree on the journey. The horse will be left in the post quartermaster's hands for *recuperation*, and finally the troop commander, who knows he may not get another in his place for a couple of years, or even more, will generally take the horse if he can be used at all, and will simply "growl."

Purchases from a contractor, by a *board of officers*, which was followed for some years previous to this method, had the drawback that officers were not always selected on account of capability or experience.

The price paid per head varied so from year to year that breeders could not look for a steady market by selling to the government. Hence, the board could never tell whether it would find suitable horses in any particular locality and would often have to make unsuccessful journeys far and wide. Then the contractor, in despair because good horses were not offered, and poor ones were not accepted, would probably protest to the Quartermaster's Department that the whole business was at a standstill, that there was little prospect of ever filling the contract, owing to the extreme exactions of the board, etc.

As a result, such members as were considered too hard to satisfy would be relieved, or the board entirely dissolved, and such horses as the contractor could get would have to be accepted, whether up to the mark or not, and paid for by an officer of the Quartermaster's Department.

This way of finally *expediting* the business has evidently led to the mode of having no board at all, and accepting without question whatever horses the inspector chooses to take. Now it is evident that this is no real improvement on the board system.

While not intending to attack the skill or integrity of any of the present citizen inspectors, which would provoke controversy and involve delays, it needs no argument to prove that if a board (even if composed of cavalry officers *not* especially skilled as examiners) has to decline nearly all the horses offered by the contractor, as *not up to the standard, that certainly the same contractor cannot present any better horses to a quartermaster's agent, at the same contract price.*

The deep-seated root of the trouble lies in the fact that the type of horse wanted for cavalry is growing scarcer and scarcer in the United States.

The demands of trade are leading to the dissemination of the heavy Percheron and Clydesdale strains amongst breeders for draft purposes ; or else, attention is devoted entirely to raising trotters, that may "lower the record ;" or to thoroughbred running horses. And so far has this been carried that officers of experience declare that in many localities where suitable horses were obtainable a few years ago, the types have changed so fast and so *completely*, that horses adapted for cavalry have now almost disappeared entirely. A step in advance was begun last year (1888). A contract at Leavenworth for a number of cavalry horses provided for their inspection by a board consisting of a "commissioned officer of the army, one veterinary surgeon, and a civilian inspector." The contract price was only $121.75 for each horse delivered and accepted. It would have been easy to predict the rejection of an immense number of horses and consequent delay and trouble. Of course this method leaves the *power* in our own hands, but as long as the contract system is followed, it is almost impossible to prevent bids being made at figures so low on account of competition as to ensure just such results.

REMEDY.—That the Association urge the authorities in Washington to *abolish the contract system and to allow purchase in open market.* This would require but the change of a few words in legislative action, and there could scarcely be any objection except that it would involve a little more work and time to get the number of horses required. On the other hand, this delay would be an advantage, for some horses would be bought later on in the fiscal year, toward Winter, when prices always drop ; or, if in any particular locality there were advantages in waiting till the following Spring, this could be done. The horses would then be shipped to the extreme southern posts at a colder season and arrive in better condition.

If citizen inspectors select *all* the horses required, they could certainly do better, and would, perhaps, be pleased and stimulated to greater exertions if allowed to buy in open market. If I am not mistaken, there is at least one inspector who is employed permanently. It would be very little additional expense to permit him to travel constantly, picking up a good horse here and there right on the farm, and have it shipped from the nearest railroad station to any point at which an officer of the Quartermaster's Department is stationed. The extra cost of sending an individual horse or a few horses would not be very much greater than by the car-load, and would be more than covered by the cheaper first cost of the animals.

The foregoing remedy is suggested as what can be done without incurring the opposition of the Quartermaster's Department—for this would still leave the purchase entirely in their hands, horses being bought by their own employés or by officers of that department, and handled until transferred to a troop entirely by that department.

I agree fully with Major Sumner in his remarks on Captain Harris' article in the Journal, March, 1888—that our horses should be bought by boards of cavalry officers of experience, and that every officer on the board should "sign the report of inspection and be held responsible for each horse."

But, as he also says: "Neglect to perform this duty properly on the part of cavalry officers has no doubt caused the framing of the law now in force." At all events, it was partly from this and from the other causes already noticed, that caused the system to be changed. If a board, composed as has been mentioned at Leavenworth, is always convened we can hardly ask more as long as the contract system is followed. There are, however, some other plans which I would urge the Association to consider.

PARTIAL REMEDIES.—As in case of entire purchase by citizen inspectors, let the "open market system" be followed, but in this scheme endeavor to get the Quartermaster General to allot only *part* of the available funds to pay for horses selected by his agents, and then send a part to regimental or post quartermasters specially for disbursement for horses that may be purchased by troop commanders or by boards of officers.

At certain posts, in this way, there would gradually get to be a steady supply of horses brought in from the neighborhood each year, as it became known that the government would purchase at a good figure, and that the money would be paid at once.

To encourage farmers and breeders, the price paid at a western post for a horse accepted there should be higher than in Illinois or Missouri, for the government would have saved the transportation expenses.

At other posts where there was no likelihood of acceptable horses being brought in for sale, it would be quite practicable to permit troop commanders in the Fall, after target, drill and manœuver seasons are ended, to travel more or less extensively through certain likely districts and draw checks on the post quartermaster when proper horses were found. In cases where by December 1st there was still money in the latter's hands, which it had been impossible to disburse judiciously, there would yet be plenty of time to return it to department or division headquarters, or to the Quartermaster General, and have it used in better localities early in the Winter when owners will sell the cheapest.

One plan proposed, of a depot in charge of selected cavalry officers, where all newly-purchased horses shall be sent to be trained and broken and then sent to troops colored and matched, we can hardly expect to inaugurate without encountering opposition from the Quartermaster's Department. It can be gradually worked up, possibly by using new horses in the troops at Fort Riley for a year, and drafting from there to adjacent regiments, replacing these again by new ones.

But small depots in charge of the Quartermaster's Department can be started without difficulty or antagonism; and under select officers of that corps, the money gradually obtained for increasing the size and scope of their "plant" will be greater than officers of the line could obtain.

The very *best* kind of remounts would be obtained from a government establishment, as selecting and mingling of different strains would be always in the direction of producing exactly the type desired. Possibly many officers think with Captain Harris, that it is almost useless to consider this scheme, as there is no probability of carrying it through at present; but an entering wedge in that direction will not be hard to obtain, nor interfere with the other proposed remedies. Let the Association work in unison with the authorities in Washington for just a line in the next appropriation bill providing for the purchase of a few stallions by the Quartermaster's Department.

Captain Pond or some other officer of the Quartermaster's Department at Fort Riley who would be interested in such work could make arrangements to have proper mares brought in there from the country about, or to take the stallions on short tours; satisfactory colts to be taken at certain rates.

The expense would be trifling at first; a few enlisted men from the large garrison at Fort Riley could be easily spared from certain duties to handle and care for sires and colts; and the Quartermaster General would certainly get from year to year larger sums and finally build up an establishment of which his corps and the army at large would be proud.

As only a few stallions would probably be appropriated for at first, the matter of who should buy them might well be left aside, even if some one most eminently adapted for the work did not happen to be selected by the Secretary of War at first; the main thing being to "feel our way" for a few years, until the scheme is matured enough to speak for itself by its good results. Then appropriations will be more and more freely bestowed.

The wonderful building up of our new navy bears witness that there is no hesitation in voting money for what is shown to be proper and legitimate for a nation of sixty millions of people.

To sum up: There would be then, 1. Part of the funds disbursed by the Quartermaster's Department, as at present, but in "open market." 2. Part turned over to the regimental and post quartermasters; if not used to be returned in time to be spent as the first part. 3. Gradual formation of sub-depots, also of one larger depot at Riley. 4. Beginning of a breeding farm there.

No matter what changes the future may bring in our weapons, ammunition, tactics or drill—one thing remains *constant:* The need of each trooper for a short-coupled, clean-limbed, sound, active, and nimble horse, of medium size, with enough "coarseness" to make him hardy and tough in the field, and enough good blood to render him spirited, intelligent and kind; rationally shod, so that he may belie the adage that one horse can wear out four pairs of legs; and carefully bitted, so that he understands and obeys the slightest pressure of rein or leg, and thus accords to his rider the utmost freedom in the use of his weapons.

Is not this within our reach?

